# Comparative Study of Structural Anomaly Diagnosis Based on ANN Model Using Random Displacement and Acceleration Responses with Incomplete Measurements

**DOI:** 10.3390/s22114128

**Published:** 2022-05-29

**Authors:** Zhi-Gang Ruan, Zu-Guang Ying

**Affiliations:** Department of Mechanics, School of Aeronautics and Astronautics, Zhejiang University, Hangzhou 310027, China; ruanzg@zju.edu.cn

**Keywords:** structural anomaly diagnosis, artificial neural network, random response, five-story building, incomplete measurements

## Abstract

Structural anomaly diagnosis, such as damage identification, is a continuously interesting issue. Artificial neural networks have an excellent ability to model complex structure dynamics. In this paper, an artificial neural network model is used to describe the relationship between structural responses and anomalies such as stiffness reduction due to damages. Random acceleration and displacement responses as generally measured data are used as the input to the artificial neural network, and the output of the artificial neural network is the anomaly severity. The artificial neural network model is set up by training and then validated using random vibration responses with different structural anomalies. The structural anomaly diagnosis method based on the artificial neural network model using random acceleration and displacement responses is applied to a five-story building structure under random base excitations (seismic loading). Anomalies in the structure are denoted by stiffness reduction. Structural anomaly diagnosis using random acceleration responses is compared with that using random displacement responses. The numerical results show the effects of different random vibration responses used on the accuracy of predicting stiffness reduction. The actual incomplete measurements include intensive noise, finite sampling time length, and limited measurement points. The effects of the incomplete measurements on the accuracy of predicting results are also discussed.

## 1. Introduction

Structural anomaly diagnosis (SAD) or structural damage identification (SDI) is very significant for reducing catastrophic failures and prolonging the service life of structures. Typical SAD or SDI methods, proposed by analyzing dynamic responses of engineering structures, include the local non-destructive testing-based method and the globe vibration-based method [1,2,3]. The vibration-based SAD/SDI method has not limitations such as certain detection regions and, thus, is increasingly studied. This method is based on the theory that variations in structural physical parameters such as stiffness cause variations in the modal parameters (i.e., modal frequencies, damping, and shapes) and vibration responses, and structural anomalies or damages result in variations in the structural physical parameters. Changes in the modal parameters, including the modal frequency [4,5,6,7,8], modal shape curvatures [9,10,11], modal strain energy [12,13,14,15], and modal flexibility [16], have been used to identify structural damages. However, an SDI method using an individual modal parameter may result in a mistake [17]. With the rapid development of computer technologies, a comprehensive SDI method based on artificial intelligence technology is developing, which has the advantage of using combined modal parameters for damage identification.

As one of the popular methods of artificial intelligence, classical artificial neural networks (ANNs) have been applied to SDI due to their excellent pattern recognition capability [18,19,20]. The basic idea of the ANN-based SDI method is to construct the relationship between structural parameter changes due to damages and modal parameters or vibration responses using the ANN model. The ANN model is set up by training and then used to identify damages as the output to input mode or response data. Many research works have reported using the combinations of modal parameters [21,22,23,24,25,26,27], mode shape differences [28,29], mode shape slope [30], and modal strain energy [31,32] as input to the ANN model to localize and assess damages. Ni et al. [33] pointed out that the input construction has significant influence on the performance and efficiency of an ANN-based SDI method. An integrated neural network framework was also proposed and used to infer system states [34]. These works have confirmed that ANN-based methods are powerful tools for SDI/SAD. However, accurate modal parameters, especially higher modal frequencies and shapes of complex structures, cannot be obtained actually. Actual complex structures cannot be modeled effectively by simplified systems and random excitations cannot be predicted; only structural responses can be obtained for use. Therefore, it has been proposed to extract damage features directly from structural vibration responses instead of modal parameters. That is both the relationship between structural anomaly (or damages) and modal parameters and the relationship between modal parameters and vibration responses are described by the ANN model. As usually measured data, structural acceleration responses have been used as input for the ANN model to identify damages [35,36,37]. Park et al. [38] presented a sequential damage identification method to identify beam damages based on ANN. Khodabandehlou et al. [39] adopted a convolution neural network to localize and assess damages of a bridge based on the ANN model using acceleration responses. Yu et al. [40] adopted a deep convolutional neural network to identify building structure damages using acceleration responses. However, structural vibration responses inevitably contain random components due to environmental and measurement noises. Structural displacement responses have generally less pollution than acceleration responses with derivatives under random excitations or noises with short correlation times. The response statistics of beam displacements have been used to identify damages based on ANN [41,42], but directly using the displacement responses of structures under random excitations to identify structural damages based on ANN needs to be studied further, along with the comparison between ANN-based SDI/SAD methods using displacement and acceleration responses.

In the present work, the SAD method based on the ANN model using random displacement responses is proposed and compared with that using random acceleration responses. The ANN-based method is applied to a five-story building structure under random base excitations for SAD by ANN output of anomaly severity. The effects of different random vibration responses used on the accuracy of predicting anomalies are shown by numerical results. The effects of the actual incomplete measurements, including intensive noise, finite sampling time length, and limited measurement points, are also discussed. Section 2 introduces an ANN model and the SAD method. In Section 3, the ANN-based SAD method is applied to a five-story building structure. Section 4 presents the results and discussions about the performance of the method using random displacement and acceleration responses and the effects of incomplete measurements, respectively. A conclusion is given in Section 5.

## 2. ANN Model and SAD Method

**Figure 1:**
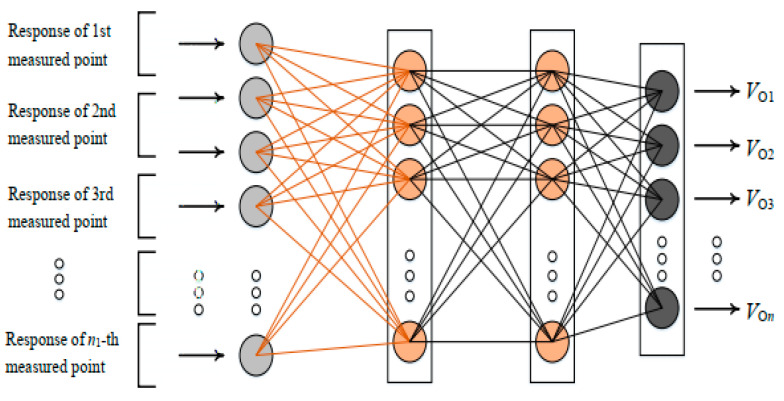
ANN architecture with two hidden layers.

The ANN model can produce meaningful solutions to complex engineering problems even if the input data have certain noises and incompleteness. Generally, the ANN model is composed of many simple computational units in layers. The classical ANN contains input, hidden, and output layers. The number of hidden layers has a variety of options. Although ANNs with many layers can represent deep circuits, training a deep network has always been somewhat of a challenge. Empirical studies have often found that deep networks generally perform no better, and often worse, than neural networks with one or two hidden layers. An ANN with many hidden layers also has a computational efficiency problem. Thus, the reasonable number of hidden layers may be a small value based on the assigned accuracy or mean square error between the aim and network outputs. Each unit in the input layer is assigned values and each unit in the output layer produces results. Units in the hidden layer are constructed to describe complex relationships by training, and then the input is connected to the output. The number of units in each layer is determined by the described problem and requirements. The number of units in a hidden layer has a variety of options. Some general rules are: (1) the number of hidden layer units is two-thirds of the number of input layers, and if this is insufficient, the number of output layer units can be added later on; (2) the number of hidden layer units should be less than twice that of the number of units in the input layer; (3) the number of hidden layer units is between the numbers of input layers and output layers [43]. An ANN with more units in the hidden layers also has a computational efficiency problem. Thus, the reasonable number of hidden layer units may be a small value based on the assigned accuracy or mean square error between the aim and network outputs under the general rules. Units between adjacent layers are connected by activation functions with certain weights [44,45]. An ANN architecture with two hidden layers is shown in Figure 1, which is used in this study.

A back-propagation algorithm (BPA) can be applied to train networks to form a mature ANN model. The mean square error (MSE), as a BPA performance criterion, is calculated by the difference in the network and aim outputs. The Adam algorithm can be used for the BPA to minimize the MSE [46]. The MSE [47,48] is expressed as

(1) $$\mathrm{MSE}=\frac{1}{n}\sum_{i=1}^{n}{\left(V_{T}{}_{i}-V_{O}{}_{i}\right)}^{2},$$

where *n* is the output number, *V*_T*i*_ is the aim output, and *V*_O*i*_ is the network output.

An ANN model is used to describe the relationship between structural responses and anomalies such as damages due to stiffness reduction. Random displacement and acceleration responses as general measured data are respectively used as the input to the ANN, and the output of the ANN is the anomaly severity. The ANN model is set up by training and then validated using random vibration responses with different structural anomalies. The ANN-based SAD method can directly use responses to assess and localize the anomalies of structures under random excitations. Structural anomaly-sensitive features can be extracted automatically from raw random acceleration and displacement responses in real time.

## 3. ANN-Based SAD for Building Structure

**Figure 2:**
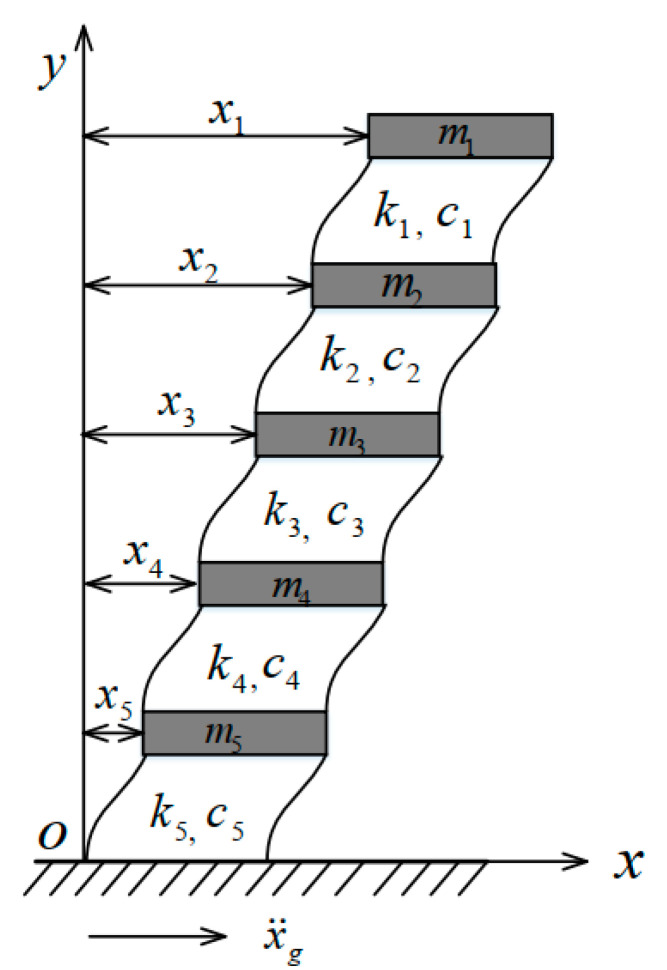
Five-story building model.

The SAD method based on the ANN model using random displacement and acceleration responses was applied to identify stiffness reduction due to damages in a building structure under random excitations. In a numerical study, a five-story building structure [36] was considered, as shown in Figure 2. The structure was modeled as a ‘shear building’ with five degrees of freedom (floor translations) and was subjected to random base acceleration excitation ${\ddot{x}}_{g}$. The excitation was a zero-mean random process and its power spectral density is described by [49]

(2) $$S_{{\ddot{x}}_{g}}(\omega )=\frac{1+4\zeta_{g}^{2}{\left(\omega /\omega_{g}\right)}^{2}}{{\left[1-{\left(\omega /\omega_{g}\right)}^{2}\right]}^{2}+4\zeta_{g}^{2}{\left(\omega /\omega_{g}\right)}^{2}}S_{0},$$

where *ω* is dimensionless frequency, the dimensionless excitation intensity *S*_0_ = 1, the frequency constant *ω*_g_ = 5π rad/s, and the damping constant *ζ_g_* = 0.6. The Kanai–Tajimi spectrum (2) is commonly used to represent random earthquake excitations, where constants are determined by ground characteristics. The structural mass, stiffness, and damping coefficients of the *i*-th story (*i* = 1,2, …, 5) are *m_i_* = 500 kg, *k_i_* = 120 kN/m, and *c_i_* = 1.0 kN·s/m, respectively. The structural vibration equation can be expressed in the matrix form

(3) $$M\ddot{X}+C\dot{X}+KX=-ME\cdot {\ddot{x}}_{g},$$

where displacement vector **X**, mass matrix **M**, damping matrix **C,** and stiffness matrix **K** are

(4) $$X=\left\{\begin{array}{c} x_{1}\\ x_{2}\\ x_{3}\\ x_{4}\\ x_{5} \end{array}\right\},\;M=\left[\begin{array}{ccccc} m_{1} & 0 & 0 & 0 & 0\\ 0 & m_{2} & 0 & 0 & 0\\ 0 & 0 & m_{3} & 0 & 0\\ 0 & 0 & 0 & m_{4} & 0\\ 0 & 0 & 0 & 0 & m_{5} \end{array}\right],\;C=\left[\begin{array}{ccccc} c_{1} & -c_{1} & 0 & 0 & 0\\ -c_{1} & c_{1}+c_{2} & -c_{2} & 0 & 0\\ 0 & -c_{2} & c_{2}+c_{3} & -c_{3} & 0\\ 0 & 0 & -c_{3} & c_{3}+c_{4} & -c_{4}\\ 0 & 0 & 0 & -c_{4} & c_{4}+c_{5} \end{array}\right],\;K=\left[\begin{array}{ccccc} k_{1} & -k_{1} & 0 & 0 & 0\\ -k_{1} & k_{1}+k_{2} & -k_{2} & 0 & 0\\ 0 & -k_{2} & k_{2}+k_{3} & -k_{3} & 0\\ 0 & 0 & -k_{3} & k_{3}+k_{4} & -k_{4}\\ 0 & 0 & 0 & -k_{4} & k_{4}+k_{5} \end{array}\right].$$

Unit vector **E** = [1, 1, 1, 1, 1]^T^, and *x_i_* is the *i*-th floor displacement relative to the base.

The displacement and acceleration responses of the structure with various stiffness reductions due to damages are obtained by Equation (3), where the random excitation samples are generated based on the power spectral density (2). The structural responses are used as the input and the corresponding relative reductions in stiffness are used as the output to train the ANN model. The relative reduction in structural stiffness is defined as

(5) $$\gamma_{i}=1-\frac{k_{i,d}}{k_{i,u}},$$

where *k_i,u_* is the structural stiffness of the *i*-th story for the normal case and *k_i,d_* is the structural stiffness of the *i*-th story for the anomaly case (*i* = 1,2, …, 5).

**Figure 3:**
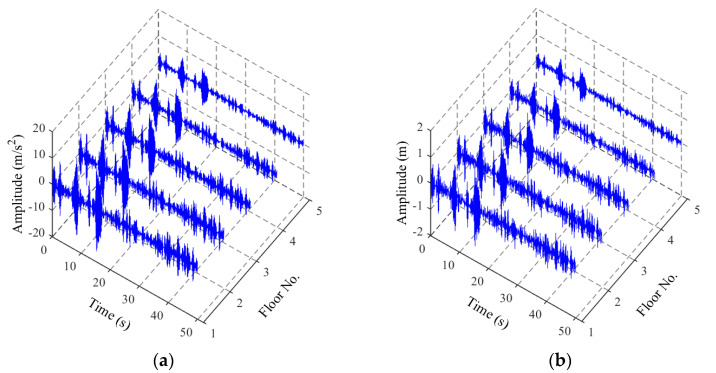
Raw vibration responses in time domain with 20 dB SNR: (**a**) acceleration responses; (**b**) displacement responses.

Table 1 lists 26 cases with and without anomalies. The structural responses in these cases were used to train and then test the ANN model for structural anomaly identification, where the anomaly is represented by the relative stiffness reduction. Case 1 is the structural normal scenario. Cases 2–14 are the single structural stiffness reductions at different stories. Cases 15–26 are the multiple structural stiffness reductions at different stories. For each condition scenario, 124 samples of random excitation produced by Equation (2) were used to obtain structural responses. A total of 3224 groups of samples were collected and divided into 124 subgroups. Then, 99 subgroups were used to train the ANN model, and 25 subgroups were used to test the model. The statistics of the output results were used to evaluate the ANN-based method. In addition, Gaussian white noises were added to the structural responses to simulate a noisy measurement. Figure 3a,b shows examples of random acceleration and a displacement responses of the structure in anomaly Case 26 (in Table 1) with a 20 dB signal-to-noise ratio (SNR), respectively. The time interval of the samples is 0.1 s and the total time is 50 s. The ANN output was the relative stiffness reduction of every story *γ_i_* (*i* = 1,2, …, 5).

The ANN model was conducted using PyTorch (in Python). Several important parameters, including the number of hidden layer units and the learning rate, were determined in advance. In this study, the number of hidden layer units was chosen as 50, and the learning rate was set as 5 × 10^−5^ according to the MSE of the training results. The ANN model was trained based on the suitable parameters using the group data, and then the ANN model was used to predict structural anomalies and its performance was evaluated by predicting results.

## 4. Results of the Incomplete Measurements of Displacement and Acceleration Responses

The results of the performance of the ANN-based SAD method using random displacement and acceleration responses and the effects of incomplete measurements, such as intensive noise, finite sampling time length, and limited measurement points, are shown in Figure 4, Figure 5, Figure 6, Figure 7, Figure 8, Figure 9, Figure 10, Figure 11, Figure 12 and Figure 13.

### 4.1. Comparison of Results Using Random Acceleration and Displacement Responses

**Figure 4:**
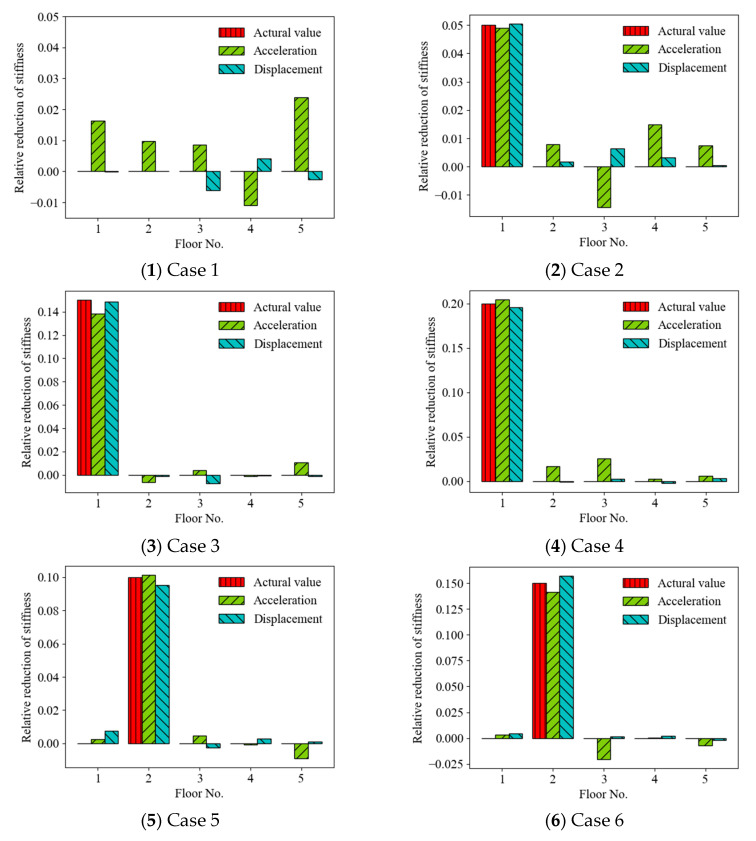

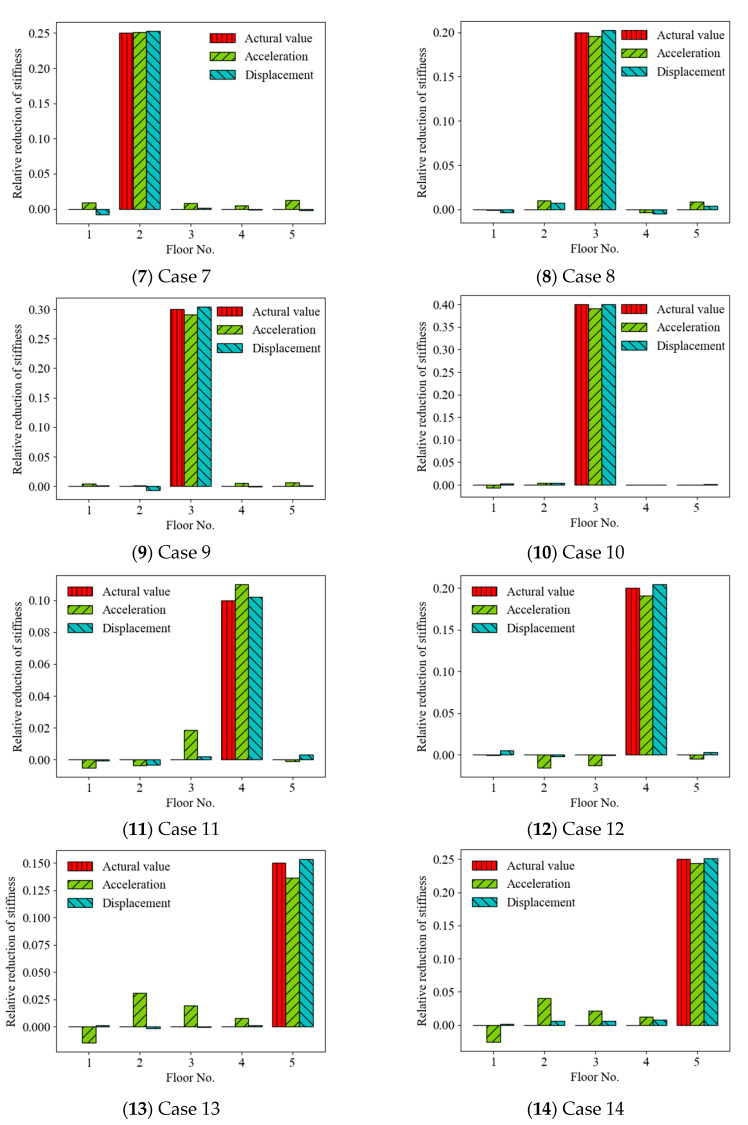

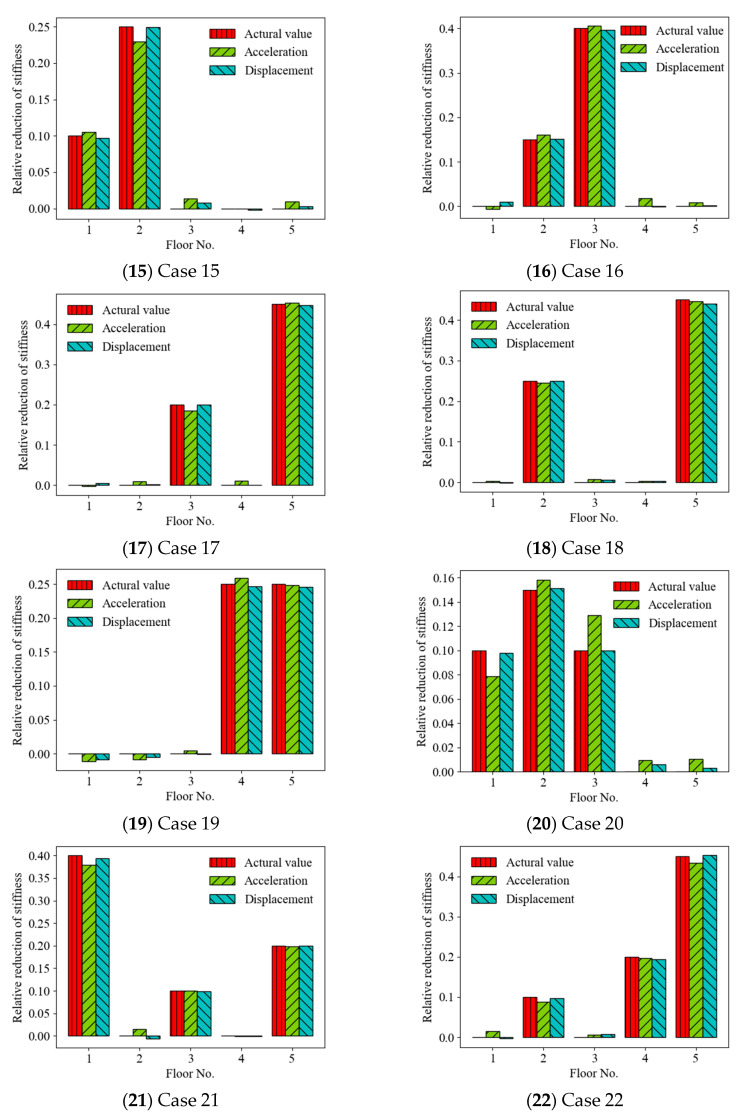

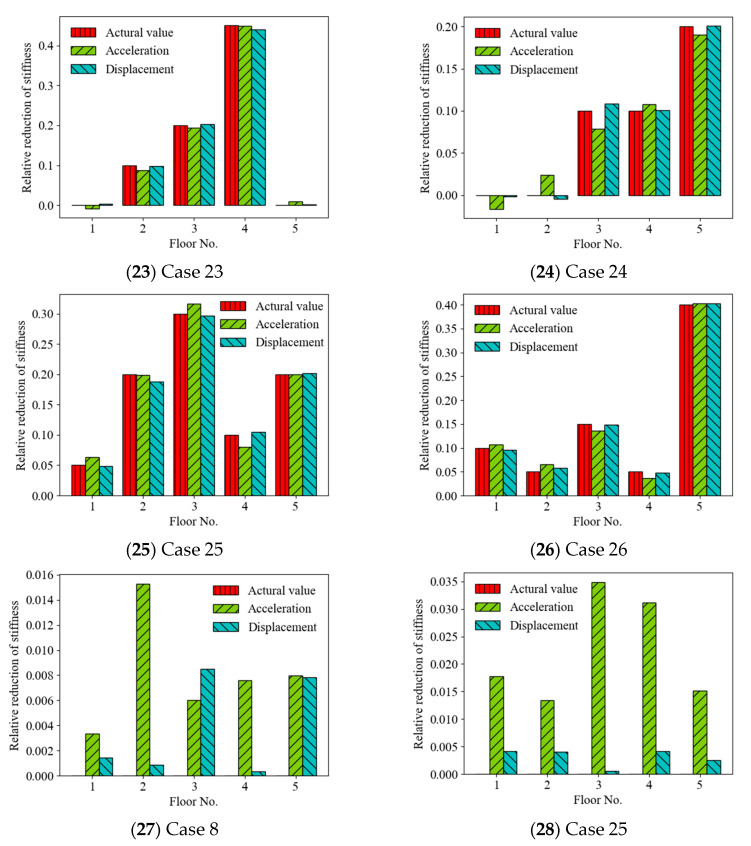
Prediction results of structural anomaly using displacement and acceleration responses as ANN input. ((**1**–**26**) Mean values of relative stiffness reduction; (**27**,**28**) standard deviations of relative stiffness reduction.

The effects of different random vibration responses (displacement and acceleration) used as the ANN input on SAD results were investigated by comparison. Figure 4 shows the prediction results of the relative reduction in structural stiffness (mean values) for various condition scenarios (Table 1) by the ANN-based SAD method using displacement and acceleration responses. The standard deviations of the relative stiffness reductions for Cases 8 and 25 are shown at the end for observation. It can be seen that the ANN-based method effectively predicted the location and severity of structural anomalies due to stiffness reduction for all cases using acceleration and displacement responses (with a 20 dB SNR) as the input. However, by comparison, the ANN-based method using displacement responses as the input roughly produced more accurate results than using acceleration responses in most cases, including Cases 1, 3, 8, 10–17, 19–22, 24, and 26. The prediction results using displacement responses had more accurate mean values and smaller standard derivations. For example, the mean value and standard derivation of the relative stiffness reduction at the first story for Case 25 were 0.0488 and 0.00418, respectively, and the confidence interval of 95% was [0.0471, 0.0504] using displacement responses, but the corresponding values using acceleration responses were 0.0634, 0.0178, and [0.0564, 0.0704], respectively. For the relative stiffness reduction at the third story of Case 8, the confidence interval of 95% was [0.199, 0.206] using displacement responses and [0.193, 0.198] using acceleration responses.

### 4.2. Effect of Noise Intensity

**Figure 5:**
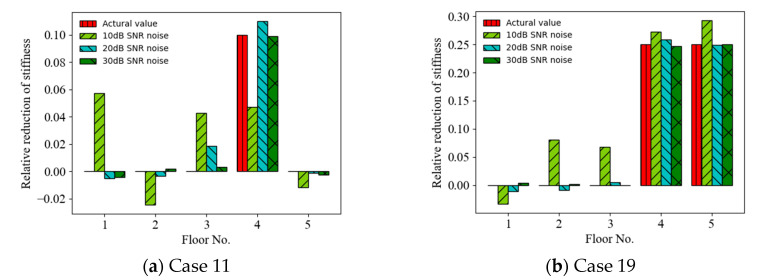
Mean prediction results of structural anomaly using acceleration responses as ANN input for different noise intensities.

**Figure 6:**
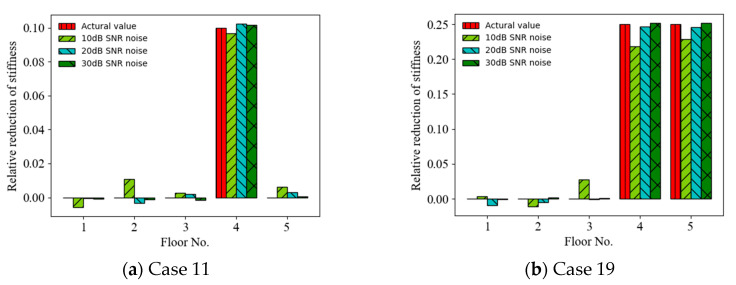
Mean prediction results of structural anomaly using displacement responses as ANN input for noise intensities.

**Figure 7:**
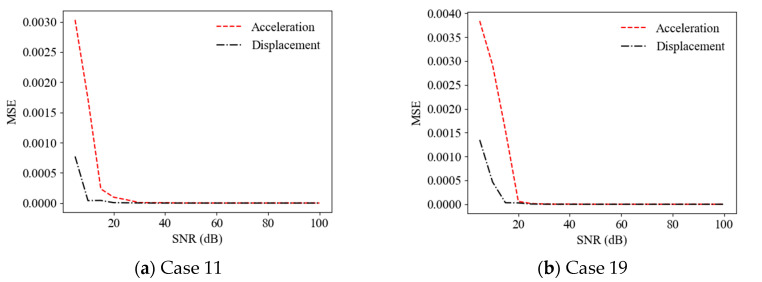
MSEs of prediction values versus SNRs using acceleration and displacement responses as ANN input.

The acceleration and displacement responses with different SNRs (representing different noise intensities) were considered as incomplete measurements, and the effect of noise intensity on the accuracy of the ANN-based SAD method was investigated. Figure 5 shows a comparison of the prediction results (mean values of relative stiffness reduction) (Cases 11 and 19) using acceleration responses as the ANN input for different SNRs. It can be observed that the accuracy of the prediction results increased with the SNR from 10 dB to 30 dB. For example, the MSEs of the prediction values are 2.92 × 10^−^^3^, 5.93 × 10^−^^5^, and 4.94 × 10^−^^6^ for SNR = 10, 20, and 30 dB, respectively, for Case 19. Figure 6 shows a comparison of the prediction results (Cases 11 and 19) using displacement responses as the ANN input for different SNRs. It can also be observed that the accuracy of the prediction results increased with the SNR from 10 dB to 30 dB. For example, the MSEs of the prediction values are 4.03 × 10^−^^5^, 6.09 × 10^−^^6^, and 1.57 × 10^−6^ for SNR = 10, 20, and 30 dB, respectively, for Case 11. Figure 7 shows the MSEs dependent on the SNR for different response inputs in Cases 11 and 19. The MSE initially decreased and then remained stable as the SNR increased. The accuracy of the prediction results using displacement responses as the ANN input was better than that using acceleration responses for smaller SNRs (e.g., less than 20 dB). Thus, the ANN-based SAD method using displacement responses as the ANN input had better robustness [50].

### 4.3. Effect of Sampling Time Length

**Figure 8:**
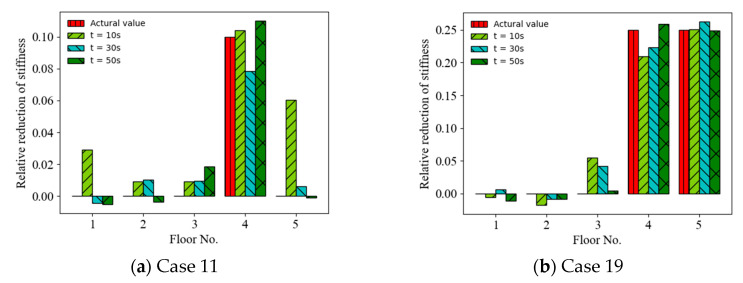
Mean prediction results of structural anomaly using acceleration responses with different time lengths as ANN input.

**Figure 9:**
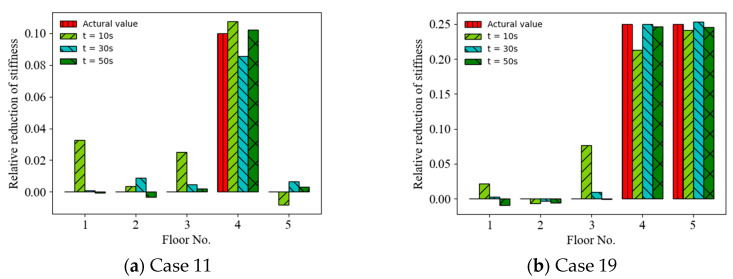
Mean prediction results of structural anomaly using displacement responses with different time lengths as ANN input.

**Figure 10:**
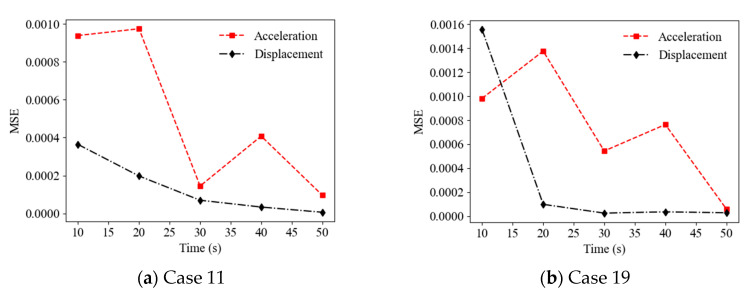
MSEs of prediction values versus time length using acceleration and displacement responses as ANN input.

The random vibration responses with diffident finite sampling time lengths were considered and the effect of the sampling time length on the accuracy of the ANN-based SAD method was investigated. Figure 8 shows a comparison of the prediction results (mean values of relative stiffness reduction) (Cases 11 and 19) using acceleration responses with different sampling time lengths as the ANN input (with a 20 dB SNR). It can be observed that the accuracy of the prediction results roughly increased with the sampling time length from 10 s to 50 s. For example, the MSEs of the prediction values are 9.83 × 10^−4^, 5.44 × 10^−^^4^, and 5.93 × 10^−^^5^ for the time lengths of 10 s, 30 s, and 50 s, respectively, in Case 19. Figure 9 shows a comparison of the prediction results (mean values of relative stiffness reduction) (Cases 11 and 19) using displacement responses with different sampling time lengths as the ANN input (with a 20 dB SNR). It can be observed that the accuracy of the prediction results also increased with the sampling time length. For example, the MSEs of prediction values are 3.64 × 10^−4^, 6.95 × 10^−^^5^, and 6.09 × 10^−^^6^ for the time lengths of 10 s, 30 s, and 50 s, respectively, in Case 11. However, the accuracy of the prediction results using displacement responses as the ANN input was better than that using acceleration responses for times longer than 15 s, as shown in Figure 10.

### 4.4. Effect of Limited Measurement Points

**Figure 11:**
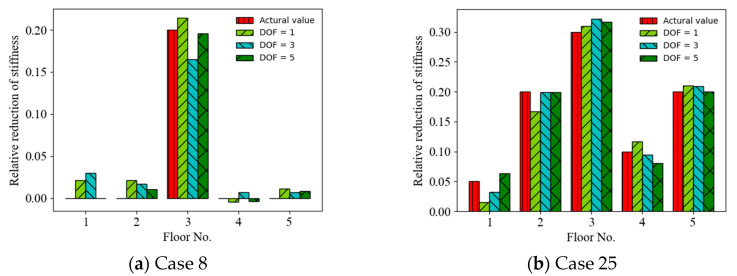
Prediction results of structural damage using acceleration responses from different measurement points as ANN input.

**Figure 12:**
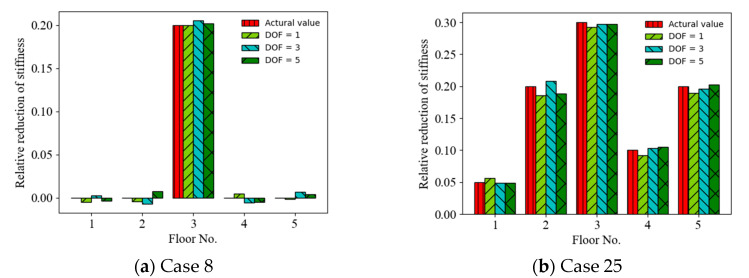
Prediction results of structural damage using displacement responses from different measurement points as ANN input.

**Figure 13:**
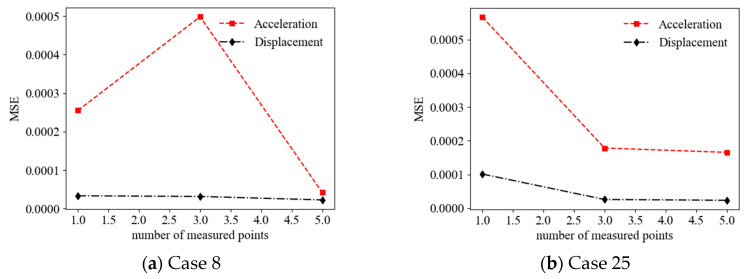
MSEs of prediction values versus number of measurement points using acceleration and displacement responses as ANN input.

The effect of the random vibration responses measured from the limited measurement points on the accuracy of the ANN-based SAD method was investigated. Three cases of limited measurement points were considered as follows: (1) the random vibration responses were measured from one measurement point (first floor) (DOF = 1); (2) the random vibration responses were measured from three measurement points (first, third, and fifth floors) (DOF = 3); and (3) the random vibration responses were measured from five measurement points (all floors) (DOF = 5). Figure 11 shows a comparison of the prediction results (mean values of relative stiffness reduction) (Cases 8 and 25) using the acceleration responses measured from different measurement points as the ANN input (with a 20 dB SNR). It can be observed that the accuracy of the prediction results roughly increased with the number of the limited measurement points from 1 DOF to 5 DOF. For example, the MSEs of the prediction values are 2.55 × 10^−4^, 4.98 × 10^−^^4^, and 4.18 × 10^−^^5^ for 1, 3, and 5 measurement points, respectively, in Case 8. Figure 12 shows a comparison of the prediction results (mean values of relative stiffness reduction) (Cases 8 and 25) using displacement responses measured from different measurement points as the ANN input (with a 20 dB SNR). It can be observed that the accuracy of the prediction results also increased with the number of limited measurement points from 1 DOF to 5 DOF. For example, the MSEs of the prediction values are 1.01 × 10^−^^4^, 2.56 × 10^−^^5^, and 2.32 × 10^−^^5^ for 1, 3, and 5 measurement points, respectively, in Case 25. The accuracy of the prediction results using displacement responses as the ANN input is better than those using acceleration responses for different numbers of measurement points, as shown in Figure 13.

## 5. Conclusions

An SAD method based on the ANN model using random displacement responses was proposed and compared with that using random acceleration responses. The ANN-based method was applied to a five-story building structure under random base excitations for SAD. Structural displacement and acceleration responses were used as the input to the ANN model and the output was the anomaly severity represented by the relative stiffness reduction. The effects of different random vibration responses used on the accuracy of the prediction of stiffness reduction are shown by numerical results and compared. The effects of the actual incomplete measurements, including intensive noise, finite sampling time length, and limited measurement points, on the accuracy of the prediction results using the ANN-based SAD method were investigated. Several meaningful results for the ANN-based SAD method were obtained as follows:

(1) Using raw acceleration or displacement responses as the input to the ANN model was more effective for SAD, and the ANN-based method using random displacement responses as the ANN input was better than that using random acceleration responses;
(2) The accuracy of the prediction results on structural anomalies increased with the response SNR, e.g., from 10 dB to 30 dB, and the results using random displacement responses as the ANN input were more accurate than those using random acceleration responses for smaller SNR (e.g., less than 20 dB); thus, the ANN-based SAD method using displacement responses as the ANN input had better robustness;
(3) The accuracy of the prediction results on structural anomalies increased with the sampling time length of random vibration responses (for certain short time lengths), and the results using random displacement responses as the ANN input were more accurate than those using random acceleration responses (e.g., for sampling times longer than 15 s);
(4) The accuracy of the prediction results on structural anomalies roughly increased with the number of limited measurement points of random vibration responses, and the results using random displacement responses as the ANN input were more accurate than those using random acceleration responses for different numbers of measurement points.

The above results are valuable for utilizing ANN with random vibration responses as the input and anomaly severity as the output to identify structural anomalies. The future work will be to incorporate the deep learning technique for improving the method performance, including robustness and resilience [51], and then to find suitable applications.

## Figures and Tables

**Table 1:** Condition scenarios of building anomalies due to stiffness reduction.

Case No.	Anomaly Location (Story No.)	Anomaly Severity (%)	Case No.	Anomaly Location (Story No.)	Anomaly Severity (%)
1	No	0	14	5	25
2	1	5	15	1, 2	10, 25
3	1	15	16	2, 3	15, 40
4	1	20	17	3, 5	20, 45
5	2	10	18	2, 5	25, 45
6	2	15	19	4, 5	25, 25
7	2	25	20	1, 2, 3	10, 15, 10
8	3	20	21	1, 3, 5	40, 10, 20
9	3	30	22	2, 4, 5	10, 20, 45
10	3	40	23	2, 3, 4	10, 20, 45
11	4	10	24	3, 4, 5	10, 10, 20
12	4	20	25	1, 2, 3, 4, 5	5, 20, 30, 10, 20
13	5	15	26	1, 2, 3, 4, 5	10, 5, 15, 5, 40

## Data Availability

Not applicable.
